# Carcinogenic risk of human papillomavirus (HPV) genotypes and potential effects of HPV vaccines in Korea

**DOI:** 10.1038/s41598-019-49060-w

**Published:** 2019-08-29

**Authors:** Eunhyang Park, Ji-Ye Kim, Sangjoon Choi, Dae Shick Kim, Young Lyun Oh

**Affiliations:** 1 Department of Pathology and Translational Genomics, Samsung Medical Center, Sungkyunkwan University School of Medicine, Seoul, Korea; 2 Department of Pathology, Yonsei University College of Medicine, Seoul, Korea; 3 Department of Pathology, Ilsan Paik Hospital, Inje University, Goyang, Korea

**Keywords:** Cancer epidemiology, Cancer screening, Cervical cancer

## Abstract

This study investigated the distribution of HPV types in Korean women and evaluated the carcinogenic risk of individual HPV types and the potential effects of HPV vaccines. A total of 4,081 HPV-positive samples between 2014 and 2017 were included. The most prevalent genotypes were HPV 16, 58, 68, and 56. Among them, HPV 16 was significantly higher in high-grade squamous intraepithelial neoplasia or worse (HSIL+ ) group. In cytologically evaluating the risk for HSIL+ by individual HPV types, HPV 16 was associated with the highest risk of HSIL+ (OR = 10.82; 95% CI: 7.93–14.77), followed by HPV 33, 31, 52, 18, 58, 51, and 35, in descending order (OR = 3.50 [type 33] to 2.62 [type 35]). Among those types, HPV 16, 18, 31, 33, and 58 were also significantly associated with HSIL+ on histologic evaluation. The analysis of the HPV subgroups covered by the different vaccines revealed that the HPV types covered by the 9-valent vaccine had a high association with HSIL+ (OR = 4.09; 95% CI: 3.02–5.54). Our findings highlight the different carcinogenic risks posed by the high risk HPV genotypes and the positive potential effects of the 9-valent HPV vaccine in reducing HPV-associated cervical cancer in Korea.

## Introduction

Human papillomaviruses (HPVs) are a well-known cause of cervical intraepithelial neoplasia (CIN) and invasive cervical cancer^1^. Of the more than 200 identified HPV types^2^, approximately 40 infect the genital area^3^. Of those, 13–15 high risk (HR) HPV types are known to be carcinogenic^4^. According to worldwide data, HPV 16 and 18 account for more than 70% of cervical cancers, and six other types (HPV 31, 33, 35, 45, 52, and 58) account for an additional 20%^5–7^. However, the contribution of specific HPV types to cervical cancer differs by region, possibly because of geographic variations in HPV type-specific prevalence in different populations^8,9^. For example, the prevalence of HPV 16 and 18 is relatively low in East Asia in both cervical cancer patients and healthy women compared to the worldwide prevalence^5,8,10^. Therefore, it is important to understand the prevalence of specific HPV types on a population-specific basis when developing strategies to prevent cervical cancer.

Three types of vaccines are currently available to prevent cervical cancer: 2-valent (Cervarix, GlaxoSmithKline, Rixensart, Belgium), 4-valent (Gardasil, Merck, Whitehouse Station, NJ), and 9-valent (Gardasil 9, Merck, Whitehouse Station, NJ) vaccines. The 2- and 4-valent vaccines protect against HPV 16 and 18, which are the two major carcinogenic types, and the 9-valent vaccine further protects against HPV 31, 33, 45, 52, and 58. Although these vaccines are expected to address approximately 70% and 90% of cervical cancers, respectively^5,6^, their preventive effects need to be evaluated on a regional basis.

The risk that an HPV infection will progress to cervical cancer varies according to the HPV genotype^4,7^. The International Agency for Research on Cancer (IARC) Working Group defined HPV types as carcinogenic if their prevalence in cancers was significantly greater than that of HPV type 6, the archetype low risk (LR) HPV type, and if they were significantly more common in cancer patients than in the general population^11^. Although a number of studies have reported the distribution of HPV types in cervical cancer patients or healthy women, few studies have evaluated the carcinogenic potential of individual HPV genotypes by analyzing case-controlled differences, as in the IARC definition^12–14^. Moreover, case-control studies rarely provide statistically valid results because of the low prevalence of HPV infection in the cohort population. Therefore, it could be beneficial to estimate the association between individual HPV types and cervical cancer in an HPV-positive group. In this study, we evaluated the distribution of HPV types in Korean women and estimated the carcinogenic potential of individual HPV types by considering the association between specific HPV types and cervical cancer or precancerous lesions. In addition, we evaluated the potential effects of HPV vaccines in Korea.

## Results

### Study population and sample evaluation

Among women who underwent HPV genotyping at Samsung Medical Center between October 2014 and March 2017, we retrieved the anonymized records of patients with co-tested cervical cytology and HPV genotyping results. For the cytology specimens, Papanicolaou-stained liquid-based tests were performed with using a ThinPrep 5000 Processor (Hologic Inc., Malborough, MA) according to the manufacturer’s protocol. Pathologists diagnosed the cytology slides according to the 2001 Bethesda system for cervicovaginal cytology: negative for intraepithelial lesions or malignancy (NILM); atypical squamous cells of undetermined significance (ASCUS); low-grade squamous intraepithelial neoplasia (LSIL); atypical squamous cells-cannot exclude high-grade squamous intraepithelial neoplasia (ASC-H); high-grade squamous intraepithelial neoplasia (HSIL); squamous cell carcinoma (SCC); and adenocarcinoma (ADC)^15^. For our analysis, we used three subgroups based on the cytologic diagnoses: NILM, LSIL (including ASCUS and LSIL), and HSIL+ (including ASC-H, HSIL, SCC, and ADC).

**Figure 1:**
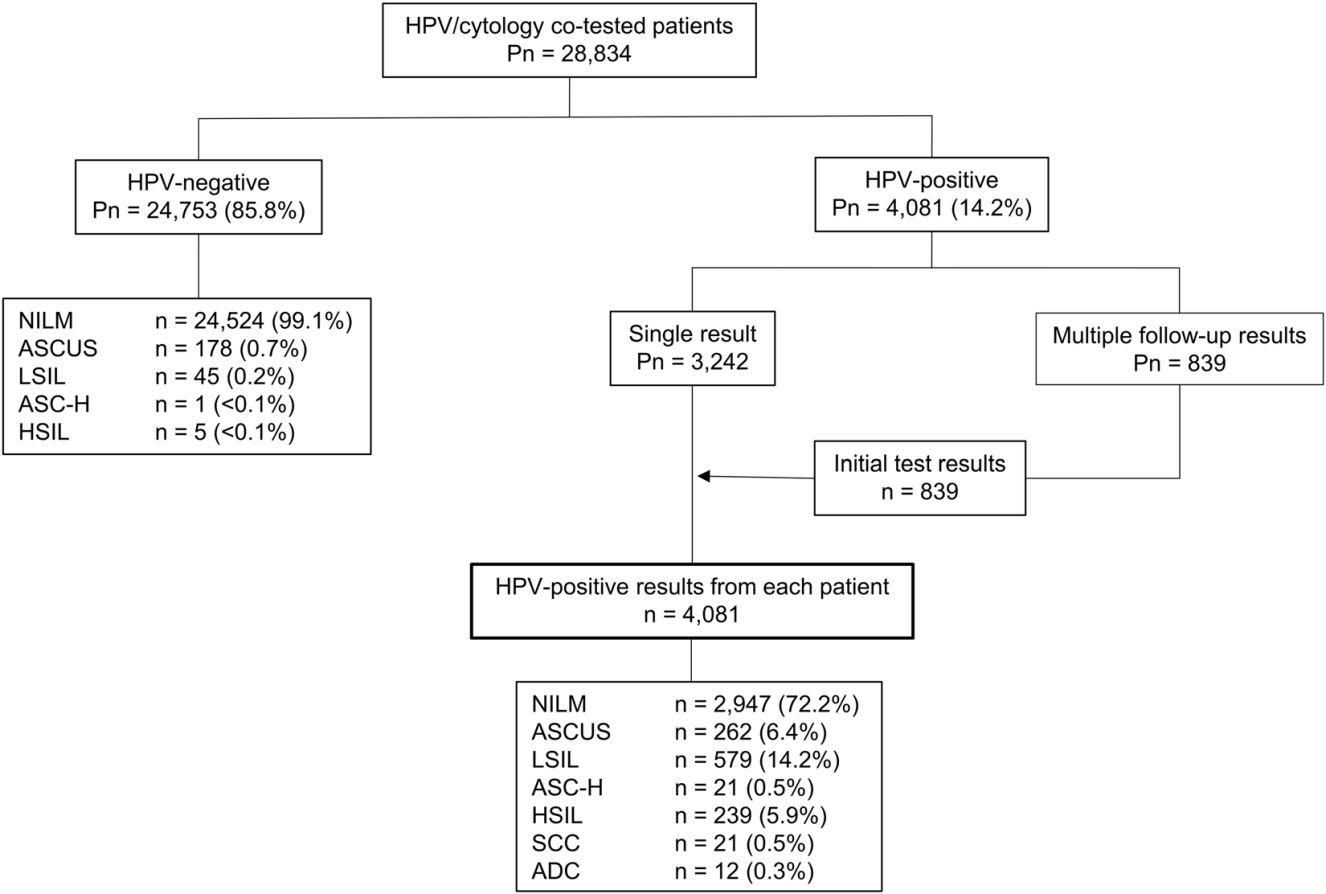
Flow diagram of patient selection. ADC = adenocarcinoma, ASC-H = atypical squamous cells-cannot exclude high-grade squamous intraepithelial neoplasia, ASCUS = atypical squamous cells of undetermined significance, HSIL = high-grade squamous intraepithelial neoplasia, LSIL = low-grade squamous intraepithelial neoplasia, NILM = negative for intraepithelial lesions or malignancy, Pn = number of patients, SCC = squamous cell carcinoma.

Of the 28,834 women with cervical cytology and HPV results, 91.5% received HPV genotyping as part of a routine health check-up, and 8.5% were referred from clinics due to abnormal cervical cytology results or other gynecologic problems. Among the 24,753 (85.8%) HPV-negative women, 0.9% had abnormal cytology results, including ASCUS (0.7%), LSIL (0.2%), ASC-H (<0.1%), and HSIL (<0.1%). Among the 4,081 HPV-positive patients, 3,242 patients had a single HPV result with a matched cytology result, and 839 patients had one or more follow-up results. We analyzed 4,081 (14.2%) HPV-positive results, including both single and initial results (Fig. 1). Among the HPV-positive patients, 596 patients (14.6%) underwent a follow-up biopsy within a year of the initial cervical pap test. The follow-up colposcopic biopsy specimens were stained with hematoxylin and eosin and diagnosed as NILM, LSIL, HSIL, SCC, or ADC. For our statistical analyses, the histologic diagnoses were also classified as NILM, LSIL, or HSIL+ (including HSIL, SCC, and ADC).

### Characteristics of the study population

The median age of the HPV-positive patients was 52 years (range: 17 to 86 years). Cytologic diagnoses were NILM in 72.2%, ASCUS in 6.4%, LSIL in 14.2%, ASC-H in 0.5%, HSIL in 5.9%, SCC in 0.5%, and ADC in 0.3%. The most common HPV type was HPV 16 (8.7%), followed by HPV 58 (7.8%), HPV 68 (7.6%), and HPV 56 (6.9%). The distributions of patient ages and HPV genotypes are summarized in Table 1.

**Table 1:** The distribution of patient ages and HPV genotypes according to cytologic diagnoses.

	n (%)				p-value
	All	NILM	LSIL	HSIL+	
All	4081	2947 (72.2)	841 (20.6)	293 (7.2)	
Age (years)					
Median (Q1–Q3)	52 (45–58)	53 (47–59)	49 (40–55)	45 (36–54)	<0.001^a^
≤34	362 (8.9)	174 (5.9)	131 (15.6)	57 (19.5)	<0.001^b,c,d^
35–44	588 (14.4)	348 (11.8)	151 (18.0)	89 (30.4)	
45–54	1528 (37.4)	1127 (38.2)	321 (38.2)	80 (27.3)	
55–64	1261 (30.9)	1025 (34.8)	192 (22.8)	44 (15.0)	
≥65	342 (8.4)	273 (9.3)	46 (5.5)	23 (7.8)	
HR HPV infection					
Yes	2400 (58.8)	1565 (53.1)	555 (66.0)	280 (95.6)	<0.001
No	1681 (41.2)	1382 (46.9)	286 (34.0)	13 (4.4)	
HR HPV genotypes					
16	356 (8.7)	186 (6.3)	64 (7.6)	106 (36.2)	<0.001^c,d^
18	140 (3.4)	96 (3.3)	24 (2.9)	20 (6.8)	0.004^c,d^
31	113 (2.8)	75 (2.5)	21 (2.5)	17 (5.8)	0.005^c,d^
33	128 (3.1)	71 (2.4)	37 (4.4)	20 (6.8)	<0.001^b,d^
35	133 (3.3)	79 (2.7)	37 (4.4)	17 (5.8)	0.002^d^
39	247 (6.1)	177 (6.0)	62 (7.4)	8 (2.7)	0.016^c^
45	62 (1.5)	45 (1.5)	16 (1.9)	1 (0.3)	0.17
51	229 (5.6)	130 (4.4)	73 (8.7)	26 (8.9)	<0.001^b,d^
52	220 (5.4)	126 (4.3)	65 (7.7)	29 (9.9)	<0.001^b,d^
56	281 (6.9)	169 (5.7)	95 (11.3)	17 (5.8)	<0.001^b,c^
58	318 (7.8)	216 (7.3)	64 (7.6)	38 (13.0)	0.003^c,d^
59	43 (1.1)	26 (0.9)	12 (1.4)	5 (1.7)	0.21
66	159 (3.9)	98 (3.3)	49 (5.8)	12 (4.1)	0.004^b^
68	309 (7.6)	228 (7.7)	63 (7.5)	18 (6.1)	0.61
Single vs. multiple HR HPV infection					
Single	2117 (51.9)	1425 (48.4)	458 (54.5)	234 (79.9)	<0.001^b,d^
Multiple	283 (6.9)	140 (4.8)	97 (11.5)	46 (15.7)	
Vaccine subgroups					
16/18	488 (12.0)	277 (9.4)	88 (10.5)	123 (42.0)	<0.001^c,d^
31/33/45/52/58	807 (19.8)	518 (17.6)	187 (22.2)	102 (34.8)	<0.001^b,c,d^
35/39/51/56/59/66/68	1293 (31.7)	867 (29.4)	338 (40.2)	88 (30.0)	<0.001^b,c^
LR HPV genotypes					
6	96 (2.4)	64 (2.2)	27 (3.2)	5 (1.7)	0.16
11	83 (2.0)	52 (1.8)	30 (3.6)	1 (0.3)	0.001
34	74 (1.8)	52 (1.8)	20 (2.4)	2 (0.7)	0.16
40	185 (4.5)	135 (4.6)	43 (5.1)	7 (2.4)	0.15
42	196 (4.8)	135 (4.6)	57 (6.8)	4 (1.4)	0.001
Others	1238 (30.3)	1035 (35.1)	194 (23.1)	9 (3.1)	<0.001

^a^Significant differences in Dunn’s test (α = 0.05) were found between the NILM and LSIL groups, the LSIL and HSIL+ groups, and the NILM and HSIL+ groups. ^b^A significant p-value after Bonferroni correction (<0.0167) was found between the NILM and LSIL groups. ^c^A significant p-value after Bonferroni correction (<0.0167) was found between the LSIL and HSIL+ groups. ^d^A significant p-value after Bonferroni correction (<0.0167) was found between the NILM and HSIL+ groups. HR = high risk, HSIL+  = high-grade squamous intraepithelial neoplasia or worse, LR = low risk, LSIL = low-grade squamous intraepithelial neoplasia, NILM = negative for intraepithelial lesions or malignancy.

Of all HPV-tested women, 5,262 (18.2%) received one or more follow-up tests. Of them, 3,745 women were HPV-negative and 1,517 patients were HPV-positive in their initial tests. Among initially HPV-positive patients, 839 patients (55.3%) were also HPV positive in their follow-up tests, whereas HPV infection of the remaining 678 patients (44.7%) had been cleared. In addition, cytologic subgroups of 209 patients (16.7%) had changed in their follow-up results (Supplementary Table S1).

### Distribution of HPV types according to cytologic subgroups

The NILM cytology group showed a high prevalence of HPV 68 (7.7%), HPV 58 (7.3%), and HPV 16 (6.3%). In the LSIL group, HPV 56 was the most common HPV type (11.3%), followed by HPV 51 (8.7%), HPV 52 (7.7%), and HPV 58 and 16 (7.6%). In the HSIL+ group, HPV 16 (36.2%) was most frequently identified, followed by HPV 58 and 52 (13.0% and 9.9%, respectively). The proportion of HR HPV infections increased significantly moving through the NILM, LSIL, and HSIL+ groups, with percentages of 53.1%, 66.0%, and 95.6%, respectively (p-value < 0.05). Among the HR HPV types, HPV 16, 18, 31, 33, 35, 39, 51, 52, 56, 58, and 66 were differently distributed in the cytologic subgroups (Bonferroni’s corrected p-values < 0.0167) (Table 1).

### Carcinogenic risk of individual HR HPV types

**Figure 2:**
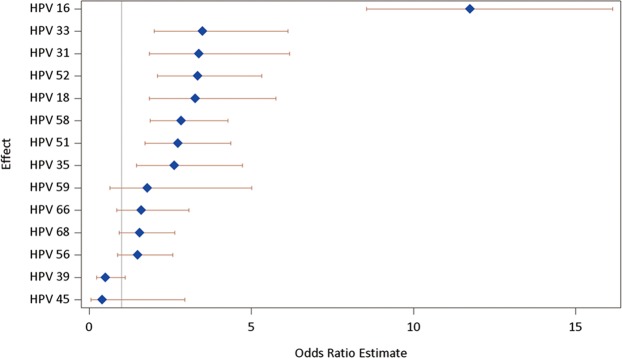
Logistic regression analyses of HPV genotypes based on cytologic results.

To assess the association of individual HPV types with HSIL+, we estimated the odds ratio (OR) of having HSIL+ (compared to NILM/LSIL) according to the individual HPV types. After adjusting for age and all other HPV types, HPV 16 was associated with the highest risk of HSIL+ (OR = 11.75; 95% CI: 8.55–16.15). In addition, HPV 33, 31, 52, 18, 58, 51, and 35, in descending order, were significantly associated with an increased risk of HSIL+ (OR ranged from 3.50 [HPV 33] to 2.62 [HPV 35]). Those data are summarized in Table 2 and visualized in Fig. 2.

**Table 2:** Multivariate logistic regression analyses of age groups and HR HPV types according to HSIL+ versus NILM/LSIL in the cytology diagnoses.

Variables	OR (95% CI)	p-value
Age group (years)		
≤34	1	
35–44	1.08 (0.72–1.61)	0.017
45–54	0.36 (0.24–0.53)	<0.001
55–64	0.23 (0.15–0.36)	0.002
≥65	0.42 (0.24–0.73)	<0.001
HR HPV types		
16	11.75 (8.55–16.15)	<0.001
18	3.26 (1.85–5.76)	<0.001
31	3.38 (1.85–6.18)	<0.001
33	3.50 (2.00–6.12)	<0.001
35	2.62 (1.46–4.72)	0.001
39	0.50 (0.23–1.11)	0.090
45	0.40 (0.05–2.95)	0.367
51	2.74 (1.71–4.37)	<0.001
52	3.34 (2.10–5.33)	<0.001
56	1.49 (0.87–2.58)	0.149
58	2.83 (1.88–4.28)	<0.001
59	1.79 (0.64–5.01)	0.268
66	1.61 (0.84–3.08)	0.151
68	1.56 (0.92–2.64)	0.102

CI = confidence interval, HR = high risk, HSIL+ = high-grade squamous intraepithelial neoplasia or worse, LSIL = low-grade squamous intraepithelial neoplasia, NILM = negative for intraepithelial lesions or malignancy, OR = odds ratio.

Among HPV-positive patients, 596 underwent a follow-up biopsy according to the “Practice guidelines for the early detection of cervical cancer in Korea”^16^: 0.7% of NILM patients, 3.1% of ASCUS patients, 55.3% of LSIL patients, 4.8% of ASC-H patients, 89.5% of HSIL patients, and 100% of SCC and ADC patients. Among patients with HSIL+ cytology, 84.3% were histologically confirmed as HSIL+. The correlations between histology and cytology diagnoses is shown in Table 3. We evaluated the association between individual HPV types and a histology-based HSIL+ result. After adjusting for age and HPV types, HPV type 16 was the most strongly associated with HSIL+ (OR = 9.22; 95% CI: 5.46–15.56), followed by HPV 31, 59, 58, 18, and 33, in descending order (OR ranged from 6.99 [HPV 31] to 3.28 [HPV 33]). Those data are shown in Table 4 and Supplementary Fig. S1.

**Table 3:** Correlations between cervical histology and cytology diagnoses.

Histologic diagnosis	NILM (n = 2)	LSIL (n = 344)	HSIL (n = 213)	SCC (n = 25)	ADC (n = 12)	Total (n = 596)
Cytology						
NILM	2 (100.0%)	18 (5.2%)	0 (0.0%)	0 (0.0%)	0 (0.0%)	20
ASCUS	0 (0.0%)	7 (2.0%)	1 (0.5%)	0 (0.0%)	0 (0.0%)	8
LSIL	0 (0.0%)	316 (91.9%)	4 (1.9%)	0 (0.0%)	0 (0.0%)	320
ASC-H	0 (0.0%)	1 (0.3%)	0 (0.0%)	0 (0.0%)	0 (0.0%)	1
HSIL	0 (0.0%)	2 (0.6%)	207 (97.2%)	5 (20.0%)	0 (0.0%)	214
SCC	0 (0.0%)	0 (0.0%)	1 (0.5%)	20 (80.0%)	0 (0.0%)	21
ADC	0 (0.0%)	0 (0.0%)	0 (0.0%)	0 (0.0%)	12 (100.0%)	12

ADC = adenocarcinoma, ASC-H = atypical squamous cells-cannot exclude high-grade squamous intraepithelial neoplasia, ASCUS = atypical squamous cells of undetermined significance, HSIL = high-grade squamous intraepithelial neoplasia, LSIL = low-grade squamous intraepithelial neoplasia, NILM = negative for intraepithelial lesions or malignancy, SCC = squamous cell carcinoma.

**Table 4:** Multivariate logistic regression analyses of HR HPV types according to HSIL+ versus NILM/LSIL in the histologic results, adjusted for age.

HPV type	OR (95% CI)	p-value
16	9.22 (5.46–15.56)	<0.001
18	3.73 (1.51–9.20)	0.004
31	6.99 (2.85–17.13)	<0.001
33	3.28 (1.54–6.96)	0.002
35	2.24 (0.94–5.33)	0.070
39	0.66 (0.28–1.58)	0.354
45	0.33 (0.04–2.64)	0.296
51	1.29 (0.68–2.43)	0.432
52	1.67 (0.85–3.25)	0.134
56	1.11 (0.57–2.16)	0.765
58	3.88 (2.12–7.10)	<0.001
59	6.59 (1.15–37.85)	0.035
66	1.03 (0.47–2.27)	0.936
68	1.29 (0.65–2.55)	0.461

CI = confidence interval, HSIL+ = high-grade squamous intraepithelial neoplasia or worse, LSIL = low-grade squamous intraepithelial neoplasia, NILM = negative for intraepithelial lesions or malignancy, OR = odds ratio.

### Potential effects of HPV vaccines

We further analyzed the HR HPV types according to vaccine coverage. HPV 16 and 18, which are covered by all HPV vaccines, were present in 12.0% of our HPV-positive patients (Table 1). HPV 31, 33, 45, 52, and 58, which are covered by the 9-valent vaccine, accounted for 19.8% of HPV-positive patients in our population. The remaining HR types (HPV 35, 39, 51, 56, 59, 66, and 68) accounted for 31.7% of our HPV-positive patients. In the HSIL+ group, the proportions of HPV 16/18 and HPV 31/33/45/52/58 were 42.0% and 34.8%, respectively. Our analysis of the cytologic results revealed that HPV 16/18 showed the highest risk for HSIL+, with an OR of 10.82 (95% CI: 7.93–14.77), followed by HPV 31/33/45/52/58 and 35/39/51/56/59/66/68, with ORs of 4.09 (95% CI: 3.02–5.54) and 1.92 (95% CI: 1.41–2.60), respectively (Table 5). Our analysis of the histologic results revealed that the ORs of HPV 16/18, HPV 31/33/45/52/58, and HPV 35/39/51/56/59/66/68 for HSIL+ were 9.46 (95% CI: 5.60–15.97), 3.93 (95% CI: 2.48–6.22), and 1.58 (95% CI: 1.00–2.49), respectively (Table 6).

**Table 5:** Multivariate logistic regression analyses of HR HPV vaccine subgroups according to HSIL+ versus NILM/LSIL in the cytology diagnoses, adjusted for age.

Vaccine subgroup	OR (95% CI)	p-value
16/18	10.82 (7.93–14.77)	<0.001
31/33/45/52/58	4.09 (3.02–5.54)	<0.001
35/39/51/56/59/66/68	1.92 (1.41–2.60)	<0.001

CI = confidence interval, HR = high risk, HSIL+ = high-grade squamous intraepithelial neoplasia or worse, LSIL = low-grade squamous intraepithelial neoplasia, NILM = negative for intraepithelial lesions or malignancy, OR = odds ratio.

**Table 6:** Multivariate logistic regression analyses of HR HPV vaccine subgroups according to HSIL+ versus NILM/LSIL in the histologic results, adjusted for age.

Vaccine subgroup	OR (95% CI)	p-value
16/18	9.46 (5.60–15.97)	0.001
31/33/45/52/58	3.93 (2.48–6.22)	<0.001
35/39/51/56/59/66/68	1.58 (1.00–2.49)	<0.001

CI = confidence interval, HR = high risk, HSIL+ = high-grade squamous intraepithelial neoplasia or worse, LSIL = low-grade squamous intraepithelial neoplasia, NILM = negative for intraepithelial lesions or malignancy, OR = odds ratio.

### Age-specific HPV prevalence and its association with cytologic diagnoses

Among all of the women in our study cohort, the overall HPV prevalence was highest (28.4%) in the ≤34 years age group, and the prevalence tended to decrease steadily with age (Supplementary Table S2 and Fig. S2). We found no discrete trends in type-specific HPV prevalence according to age. In the association between age and cytologic diagnosis, the median age of LSIL patients was 49 years, and the prevalence of LSIL showed a single peak in the 45–54 years age group. Among HSIL+ patients, the median age was 45 years, with a peak at 35–44 years followed by a decrease with age. The ages of the NILM, LSIL, and HSIL+ cytologic groups differed significantly (Bonferroni’s corrected p-value < 0.0167). Our logistic regression analyses showed that older patients (>45 years) had an inverse risk for HSIL+ (Table 2).

### Infection by multiple HR HPV genotypes

HR HPV types were co-infected in 6.9% of HPV-positive patients, with two to six concomitant HPV types (Supplementary Table S3). The percentage of multiple infections increased significantly from 4.8% in the NILM cytologic subgroup to 11.5% in the LSIL subgroup and 15.7% in the HSIL+ subgroup (p < 0.05). HPV 58 (24.7%), 16 (21.9%), and 51 (21.6%) were frequently identified as co-infections, and HPV 16–58 and HPV 33–35 were the two most common combinations of HPV types.

## Discussion

Our aim in this study was to investigate the distribution of HPV types in Korean women and evaluate the association between individual HPV types and the risk of precancerous lesions and cervical cancer. We also evaluated the potential effects of current HPV vaccines.

In HPV-positive patients, the most frequent HPV type was HPV 16, followed by HPV 58 and 68. In patients with HSIL+, HPV 16, 58, and 52 were frequently identified, with HPV 16 as the most prevalent type. Although previous studies in Korean populations have reported inconsistent results regarding the HPV type–specific distribution^17–19^, those studies did agree that, compared with the worldwide prevalence^20,21^, HPV 58 is one of the most prevalent types in Korea, with HPV 16 and 18 being relatively less prevalent. This finding implies that the 9-valent vaccine, which covers five HR HPV types in addition HPV 16 and 18, would be beneficial to use in Korea.

In this study, the proportion of HR HPV types increased significantly from the NILM to the LSIL and HSIL+ cytology groups. However, not all HR HPV types were associated with HSIL+ . The analysis of cervical cytology revealed that HPV 16, 18, 31, 33, 35, 51, 52, and 58 were significantly associated with HSIL+. Of those, HPV 16, 18, 31, 33, and 58 were also associated with HSIL+ in histologically confirmed cases. Although HPV 59 showed a significant association with HSIL+ in the histologic evaluations, only six HPV 59 positive samples were included in the histologic analysis, so that the result should be interpreted with caution. Previous studies traced the disease progression of HPV-positive NILM or LSIL cytology patients and found that HPV 16, 18, 31, 33, 35, 52, and 58 were associated with the risk for HSIL+ ^22–25^, which is consistent with our findings. Because HR HPV genotypes confer significantly different prognostic information, it might be helpful to develop guidelines for specific HPV types to effectively manage patients.

HPV 16, 18, 31, 33, and 58, which all had high carcinogenic potential in both the cytology and histology results, are covered by the 9-valent vaccine. When the risk for HSIL+ was evaluated in the HR HPV subgroups according to vaccine coverage, the HPV types (HPV 31/33/45/52/58) covered by the 9-valent vaccine were associated with twice the risk for HSIL+ than the HR HPV types (HPV 35/39/51/56/59/66/68) not covered by the vaccine. Thus, the 9-valent vaccine could be effective at preventing cervical cancer in the Korean population. In Korea, the 2-valent and 4-valent vaccines are currently included in the national immunization program as a 2-dose schedule for 12-year old girls. The 9-valent vaccine was approved by the Ministry of Food and Drug Safety in January 2016 and can be given as a non-national immunization program vaccine. Huh *et al*. reported that the 9-valent vaccine showed efficacy against HPV 31, 33, 45, 52, and 58 and similar efficacy toward HPV 6, 11, 16, and 18 as the 4-valent vaccine^26^. In addition, 2-doses of the 9-valent vaccine showed non-inferior immunogenicity to the standard 3-doses in 10–14-year-old females^27^. Although factors such as cost should certainly be considered when determining a national immunization program, implementation of the 9-valent vaccine might produce a substantial decrease in cervical cancer in Korea.

Among HPV-positive patients, the median age was youngest in the HSIL+ group, gradually increasing in the LSIL and NILM cytology groups. The prevalence of HSIL+ peaked in the 35–44 years group and decreased with age. These findings are in line with other recent studies in Korea^18,28,29^. Because women start being infected with HR HPV when they become sexually active, and HR HPV can cause HSIL in approximately 7 years, HSIL most commonly happens to women in their early 30s^30^. In addition, the age-specific prevalence of CIN is attributable to the coverage of cervical cancer screening^31,32^. Because cervical cancer screening is registered as a national cancer screening program in Korea, the screened population could receive timely therapeutic intervention, allowing the prevalence of HSIL+ to decrease in the older age groups.

This study has some limitations. First, it is a retrospective cross-sectional study. In addition, the HPV genotyping was performed with two different methods—the HPV 9G DNA Chip Test and the GeneFinder in the first and second halves of the study period, respectively. Although both methods have been validated^33–35^, no direct comparison of these two tests has been performed, so there could be discordance between them. Instead, we compared the results of the study periods covered by the different HPV genotyping methods. Of the total 28,834 cytology/HPV co-tested women, 13,697 patients (47.5%) were tested using the DNA chip and 15,137 patients (52.5%) were tested using the GeneFinder. The prevalence of HPV was 17.5% in the first period and slightly but not significantly increased to 18.3% in the second period. The prevalence of HSIL+ was 6.6% in the first period and 5.6% in the second period, while HPV 16 was identified in 1.8% and 1.5% in the first and second periods, respectively. Although the prevalence of HSIL+ and individual HPV types did differ in the two periods, the genotyping tests were performed in different populations, making it difficult to identify any significant differences between the two methods. Nonetheless, this large-scale study identified the distribution of individual HPV types in Korean women and used risk stratification to assess the carcinogenic potential of specific HPV types. In addition, we found a high correlation between cytology and histology diagnoses, indicating that our results are reliable. This study thus provides guidance for improving the HPV-related national immunization program and management guidelines for individual HPV types.

## Methods

### Ethical statement

All specimens and related data were obtained with approval from the Institutional Review Board of Samsung Medical Center (SMC 2018-07-104) with a waiver of informed consent in accordance with HHS regulation 45CFR46.116(f). All methods were carried out according to the ethical standards of the institutional and national research committees and the Declaration of Helsinki.

### HPV genotyping

HPV genotyping was performed with an HPV 9G DNA chip (Biometrix Technology Inc., Chuncheon, South Korea) or a GeneFinder HPV Liquid Bead MicroArray Genotype kit (GeneFinder; Infopia Inc., Anyang, South Korea). The HPV 9G DNA chip test was used from October 2014 to December 2015, and the GeneFinder was used from January 2016 to March 2017.

The HPV 9G DNA chip detects HPV genotypes via microarray, and the test was performed according to the manufacturer’s protocol^34^. Briefly, HPV genomic DNA was extracted from cervicovaginal cytology samples and amplified via polymerase chain reaction (PCR). The PCR product was mixed with hybridization solution and loaded on the HPV 9G DNA chip. The DNA chip was then hybridized and washed at room temperature (25 °C). The final product was automatically scanned and analyzed.

The GeneFinder is a microsphere bead–based test that is highly specific and sensitive for detecting HPV genotypes^36^. After HPV genomic DNA was extracted, the specimen was mixed with HPV type-specific polystyrene microspheres dyed with specific intensities of fluorophores. After hybridization and PCR amplification, the microspheres were streamed using Luminex technology (Luminex, Austin, TX). HPV genotypes were detected by a Luminex analyzer based on a flow cytometry method.

The HPV 9G DNA chip detects 19 HPV types (HPV 6, 11, 16, 18, 31, 33, 34, 35, 39, 40, 42, 45, 51, 52, 56, 58, 59, 66, and 68) and screens for 19 other types (HPV 3, 10, 26, 27, 32, 43, 44, 53, 54, 55, 57, 61, 62, 67, 69, 70, 71, 73, and 74) without specific typing. The GeneFinder detects 32 HPV types (HPV 6, 11, 16, 18, 26, 31, 32, 33, 34, 35, 39, 40, 42, 43, 44, 45, 51, 52, 53, 54, 55, 56, 58, 59, 62, 66, 68, 69, 70, 73, 81, and 83). We analyzed the 19 HPV types (HPV 6, 11, 16, 18, 31, 33, 34, 35, 39, 40, 42, 45, 51, 52, 56, 58, 59, 66, and 68) detected by both tests. Among them, 14 HPV types (HPV 16, 18, 31, 33, 35, 39, 45, 51, 52, 56, 58, 59, 66, and 68), including IARC Groups 1 and 2A^11^, are regarded as HR HPV, whereas HPV 6, 11, 34, 40, and 42 are regarded as LR HPV. If patients had an HPV type identified by one method but not the other, the HPV type was categorized as “other.”

### Statistical analysis

Patient ages were stratified into five subgroups: ≤34 years, 35–44 years, 45–54 years, 55–64 years, and ≥65 years. A χ^2^ test or Fisher’s exact test was used to evaluate the statistical significance of differences in categorical variables among groups. Post-hoc test was performed using Tukey’s Honestly Significant Difference test, and p-values from multiple comparisons were corrected by the Bonferroni method. Age was additionally analyzed as a continuous variable using the Kruskal-Wallis test followed by Dunn’s post-hoc test. To assess the association of HPV subtypes with HSIL+, we performed logistic regressions and calculated the OR and 95% CI. For HPV types, the OR was defined as the ratio between the odds of being in the HSIL+ group in the presence of an individual HPV type and the odds of being in the HSIL+ group in the absence of an individual HPV type. The results were visualized by forest plot using the SGPLOT procedure. For all analyses, p < 0.05 was considered statistically significant, and all analyses were two-sided. Data were analyzed using SAS version 9.4 (SAS Institute Inc., Cary, NC).

## Supplementary information

Supplementary Figure S1-S2, Table S1-S3

## Data Availability

Most data are provided in the manuscript and Supplementary Information. Other data will be available from the corresponding author upon reasonable request.
