# Supplementary material for: Carcinogenic risk of human papillomavirus (HPV) genotypes and potential effects of HPV vaccines in Korea
Source: Sci Rep. 2019 Aug 29;9:12556. doi: 10.1038/s41598-019-49060-w (PMC6715722; doi:10.1038/s41598-019-49060-w)
Supplement: Supplementary file 1 — Supplementary Figure S1-S2, Table S1-S3 [file 41598_2019_49060_MOESM1_ESM.docx]

**Supplementary information**

**Carcinogenic risk of human papillomavirus (HPV) genotypes and potential effects of HPV vaccines in Korea**

Eunhyang Park^1,2^, Ji-Ye Kim^3^, Sangjoon Choi^1^, Dae Shick Kim^1^, Young Lyun Oh^1*^

^1^Department of Pathology and Translational Genomics, Samsung Medical Center, Sungkyunkwan University School of Medicine, Seoul, Korea

^2^Department of Pathology, Yonsei University College of Medicine, Seoul, Korea

^3^Department of Pathology, National Cancer Center, Goyang, Korea

**Correspondence to:**

Young Lyun Oh, M.D., Ph.D.

Department of Pathology and Translational Genomics, Samsung Medical Center, 81 Ilwon-ro, Gangnam-gu, Seoul, 06351, Korea. Tel: +82-2-3410-2805; Fax: +82-2-3410-0025; E-mail: bijou@skku.edu

**
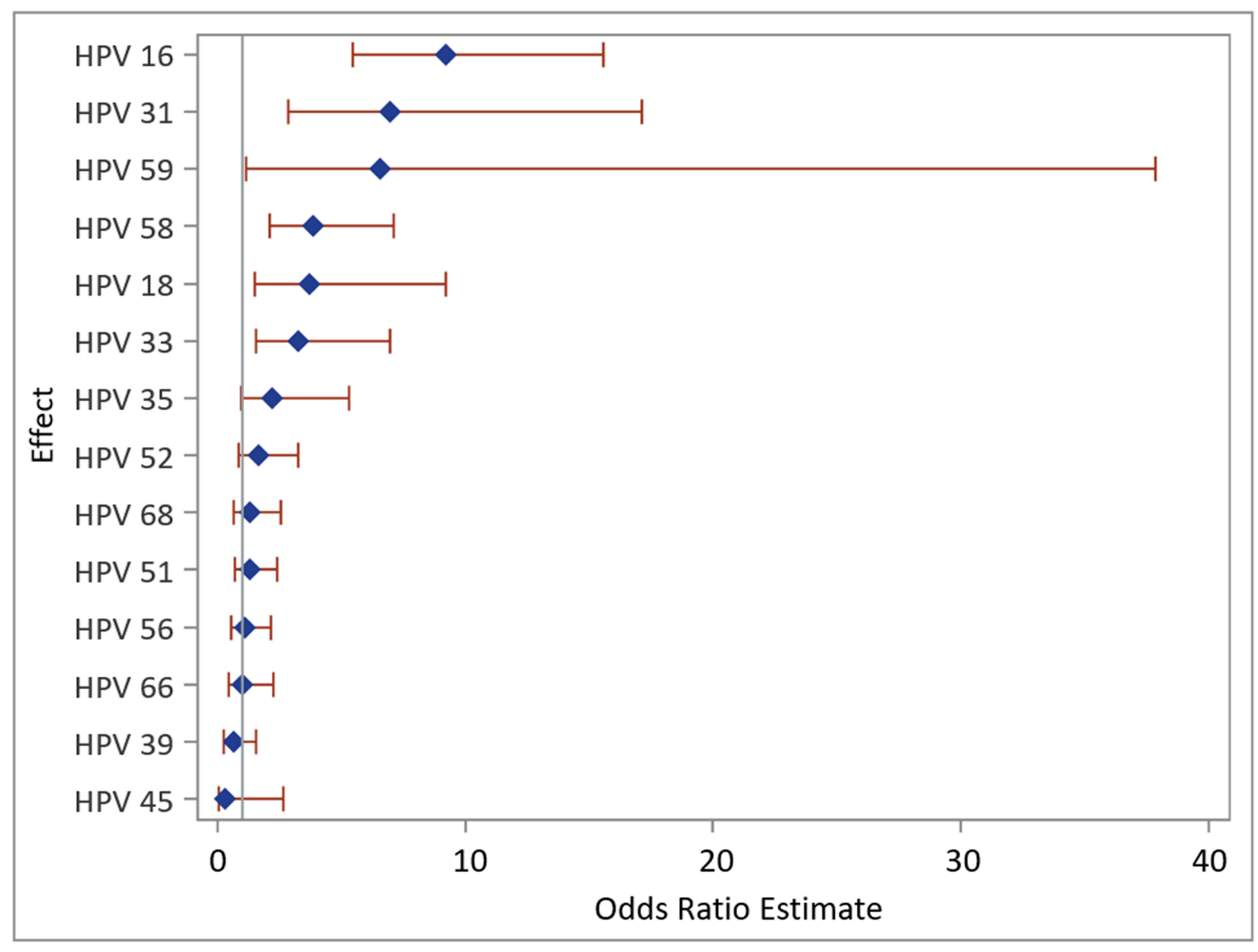
**

**Supplementary Figure S1.** Logistic regression analysis of HPV genotypes based on histologic results.

**Supplementary Figure S2.** HPV prevalence among HPV-tested women by age group.

| **Initial cytology** | **Follow-up cytology** | | | **Total** |
| --- | --- | --- | --- | --- |
|  | **NILM** | **LSIL** | **HSIL+** |  |
| NILM | 1164 (92.9%) | 72 (5.7%) | 17 (1.4%) | 1253 |
| LSIL | 88 (37.8%) | 133 (57.1%) | 12 (5.2%) | 233 |
| HSIL+ | 9 (29.0%) | 11 (35.5%) | 11 (35.5%) | 31 |
| Total | 1261 | 216 | 40 | 1517 |

**Supplementary Table S1.** Changes in cytologic subgroups of initial HPV-positive patients on follow-up.

| **Age group (years)** | **HPV positive** | **Total** |
| --- | --- | --- |
| ≤34 | 362 (28.4%) | 1274 |
| 35–44 | 588 (15.4%) | 3809 |
| 45–54 | 1528 (13.3%) | 11513 |
| 55–64 | 1261 (13.7%) | 9202 |
| ≥65 | 342 (11.3%) | 3036 |
| Total | 4081 | 28834 |

**Supplementary Table S2.** Age-specific prevalence of HPV infection in HPV-tested women.

|  | **n (%)** | | | |
| --- | --- | --- | --- | --- |
|  | **Total** | **NILM** | **LSIL** | **HSIL+** |
| Single HR HPV | 2117 (51.9) | 1425 (48.4) | 458 (54.5) | 234 (79.9) |
| Multiple HR HPV | 283 (6.9) | 140 (4.8) | 97 (11.5) | 46 (15.7) |
| 2 | 239 (5.9) | 129 (4.4) | 72 (8.6) | 38 (13.0) |
| 3 | 36 (0.9) | 7 (0.2) | 21 (2.5) | 8 (2.7) |
| 4 | 6 (0.1) | 3 (0.1) | 3 (0.4) |  |
| 5 | 1 (<0.1) | 0 (0.0) | 1 (0.1) |  |
| 6 | 1 (<0.1) | 1 (<0.1) |  |  |

**Supplementary Table S3.** The prevalence of multiple HR HPV infections according to cytologic diagnoses. HR = high risk, HSIL+ = high-grade squamous intraepithelial neoplasia or worse, LSIL = low-grade squamous intraepithelial neoplasia, NILM = negative for intraepithelial lesions or malignancy.
